# Our experience with super-thin anterolateral thigh flap for intraoral reconstruction

**DOI:** 10.1007/s10006-026-01616-7

**Published:** 2026-08-12

**Authors:** Attila Füzes, Szilárd Szanyi, Balázs Kovács, András Tarczali, Márk Huszák, Tibor Sahin-Tóth, Dániel Csizmazia, Marcell Szanyi, Dániel Végh, László Tamás, Ferenc Oberna

**Affiliations:** 1 Multidisciplinary Head and Neck Cancer Center, National Institute of Oncology, 1122 Ráth György Utca 7-9, Budapest, Hungary; 2 National Tumor Biology Laboratory, National Institute of Oncology, Budapest, Hungary; 3 Department of Morphology and Physiology, Faculty of Health Sciences, Semmelweis University, Budapest, Hungary; 4 Department of Prosthodontics, Semmelweis University, Budapest, Hungary; 5 Department of Otorhinolaryngology, Head and Neck Surgery, Faculty of Medicine, Semmelweis University, Budapest, Hungary

**Keywords:** Head and neck cancer, Free flap, Anterolateral thigh flap, Perforator Flaps, Super-thin ALT

## Abstract

**Purpose:**

According to the literature, in our country, the first choice for the reconstruction of extensive defects after the removal of locally advanced oral tumors is free flap reconstruction, which achieves the best functional outcome. We investigated whether the anterolateral thigh flap, which can be closed primarily at the donor site and is usually indicated for large or complex defects, can be adapted for thin defects, as well.

**Materials and methods:**

Within the framework of a pilot study, 10 patients who underwent radical ablative surgery for oral squamous cell carcinoma were reconstructed with an anterolateral thigh flap harvested in the superficial fascia plane. We compared our results with those of 42 oral cavity radial forearm flap reconstruction procedures.

**Results:**

Of the 10 patients who underwent reconstruction with a thinned anterolateral thigh flap seven underwent surgery for sublingual tumor and three for tongue tumors. All the flaps healed uneventfully. The average flap thickness was 9.25 mm and the average pedicle length was 11.7 cm, which was similar to that of the radial forearm flap (8.6 mm and 12.1 cm). No complications were observed at the donor site, and in every case, we were able to close the donor site primary.

**Conclusion:**

The anterolateral free thigh flap harvested in the superficial fascia plane could be an appropriate choice for functional preservation and closure of the oral cavity. Considering that it is similar to the radial forearm flap in terms of thickness and pedicle length but has a more favorable donor site morbidity, its wider use is recommended.

**Supplementary Information:**

The online version contains supplementary material available at 10.1007/s10006-026-01616-7.

## Introduction

In 2019, there were 2,958 malignant head and neck tumors were registered in our country, making it the 8th most common cancer worldwide. This year, 1171 of these patients were diagnosed with lip and oral cancer. The incidence of these tumors is twice as high in men as in women [1, 2].

Free tissue reconstruction is the first choice for restoring tissue defects after the removal of locally advanced oral tumors [3–5]. The aim of reconstructive surgery is to preserve the function of the oral cavity by preserving the function of the remaining tissues, ensuring postoperative protection, and safely closing the oral cavity from the neck, which are opened together during surgery [6–8]. In our country, the most commonly used free flap for the reconstruction of superficial oral defects is the fasciocutaneous radial forearm flap (RFF), which meets the reconstructive goals owing to its surface, thickness, and pliability. For thicker, bulkier, or more extensive defects, a musculocutaneous or perforator anterolateral thigh flap (ALT) is the appropriate choice. Our practice predominantly uses free osteoseptocutaneous fibula flap for the reconstruction of bony or composite defects. In the case of flap necrosis or vascular anomalies, a secondary repair option, mainly for women, is the musculocutaneous latissimus dorsi flap as a pedicle or free flap, whereas for men, the musculocutaneous pectoralis major flap is used as a pedicle flap.

Donor-site morbidity of free radial forearm flaps chosen to repair superficial oral defects is significant because the donor site usually has to be skin grafted. In addition, when harvesting the free radial forearm flap, the radial artery is removed along with the flap, therefore a reduction in hand grip strength and sensory disturbance of the thumb and index finger can be expected [9, 10].

For the reasons described above, we sought a reconstructive option to replace the radial forearm flap with similar characteristics and lower donor site morbidity [9–11]. After a thorough understanding of the anatomy of the perforator ALT flap, which was previously used only to restore large or thick defects without significant complications, we started using it for the reconstruction of superficial oral defects after thinning. In 1996, Kimura wrote about the concept of flap thinning [12]. The technique of thinning the anterolateral thigh flap along the superficial fascia, the so-called super-thin ALT (sALT), was first described in the literature in 2014, and the first free flap in our national literature using this technique was published by our group [13, 14].

Our hypothesis is that the super-thin ALT flap has similar reconstructive surgical characteristics to the radial forearm flap, but with a lower donor site morbidity.

## Materials and methods

The aim of this study is to compare two surgical techniques in a pilot and a retrospective comparative study. The study was approved by the institutional and national ethics committees (ETT TUKEB, Medical Research Council) (project number BMEÜ/1825–1/2022/EKU). Data for the pilot study were collected prospectively, while data for the control group were collected consecutively, retrospectively from the electronic database and medical reports.

Between 2020 and 2025 at the Multidisciplinary Head and Neck Cancer Center of the National Institute of Oncology, 52 patients underwent head and neck cancer surgery with microsurgical reconstruction in the oral cavity.

The inclusion criteria were (1) over the age of 18; (2) tongue or floor-of-mouth cancer; (3) partial glossectomy and floor-of-mouth resection; (4) reconstructed with sALT or RFF.

Ten patients underwent reconstruction with free sALT flap, and 42 patients with free RFF. Patients with incomplete data fields were filtered out during the retrospective selection process. Patients who underwent restoration with radial forearm flap larger than 80 cm2 were excluded from the study to create a similar study population. The two groups were compared in terms of tumor and flap characteristics: T stage (TNM classification of the UICC, 8th edition, 2016), flap thickness, surface, vascular pedicle length, flap harvesting time, and operation time, postoperative complications: flap necrosis, donor site complications, nasogastric (NG) tube and tracheotomy tube removal, and inpatient time. Operative time was defined as the time in minutes from the first incision to the last stitch.

Quality of life and functional outcomes were analyzed using the EORTC QLQ-C30, H&N35, and FACT-H&N questionnaires in 10 sALT and 10 RFF patients. We selected the EORTC QLQ-C30/QLQ-H&N35 and FACT-H&N because they are validated head and neck oncology patient-reported outcome measures with complementary strengths. The EORTC system combines a cancer core questionnaire with a disease-specific head and neck module, allowing assessment of both global quality of life and site-specific symptoms, whereas FACT-H&N emphasizes functional well-being and symptom burden from the patient perspective. Together, these instruments might provide a multidimensional assessment of function and quality of life after oral cancer resection and free-flap reconstruction. The questionnaires were completed before surgery and on the day of discharge.

Composite questionnaire scores were analyzed as summed ordinal outcomes. Although Shapiro–Wilk testing did not demonstrate a significant departure from normality in any of the 4 datasets for either instrument, and variance testing did not show a consistent pattern of heteroscedasticity across comparisons, nonparametric methods were selected a priori because the study variables were derived from summed ordinal questionnaire items and the sample size was limited.

The primary endpoint is whether (1) sALT is suitable for intraoral reconstruction; (2) can we recruit the sample size recommended by the power analysis?

As a secondary endpoint, (1) is there a difference between the two surgical techniques; (2) can a comparable flap be created from the thigh (sALT) as well as from the forearm (RFF); (3) is there a difference in the rate of donor site complications and in the quality of life? Data were compared using the independent t-test, Fisher’s exact test, Shapiro–Wilk test, Mann–Whitney U-test, exact Wilcoxon test. The SPSS 29 software was used for statistical analysis. Due to the small sample size and retrospective nature of this pilot study (10 vs. 42 patients), statistical adjustments for confounding factors were not feasible. No sensitivity analyses were performed for this clinical study.

### Surgical technique

**Fig. 1:**
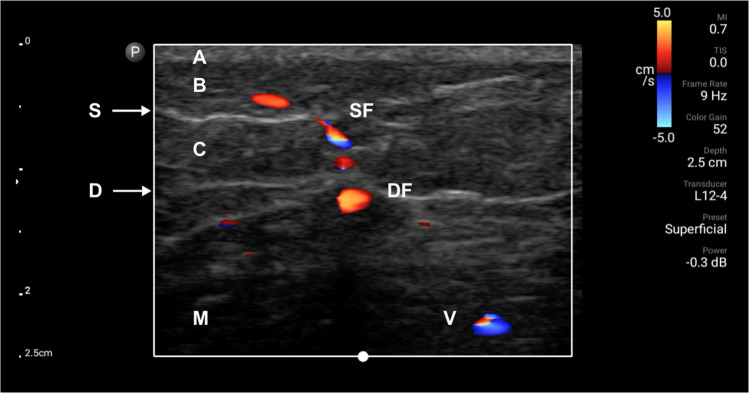
Preoperative color duplex ultrasonography from the perforator on the left thigh. A: skin; B: superficial fat layer of the subcutaneous tissue; C: deep fat layer of the subcutaneous tissue; S: superficial fascia layer of the subcutaneous tissue; D: deep fascia layer of the of the subcutaneous tissue; SF: the exact spot where the perforator pierces the superficial fascia; DF: the exact spot where the perforator pierces the deep fascia; M: vastus lateralis muscle; V: intramuscular branch of the vascular pedicle (descending branch of the lateral circumflex femoral artery)

**Fig. 2:**
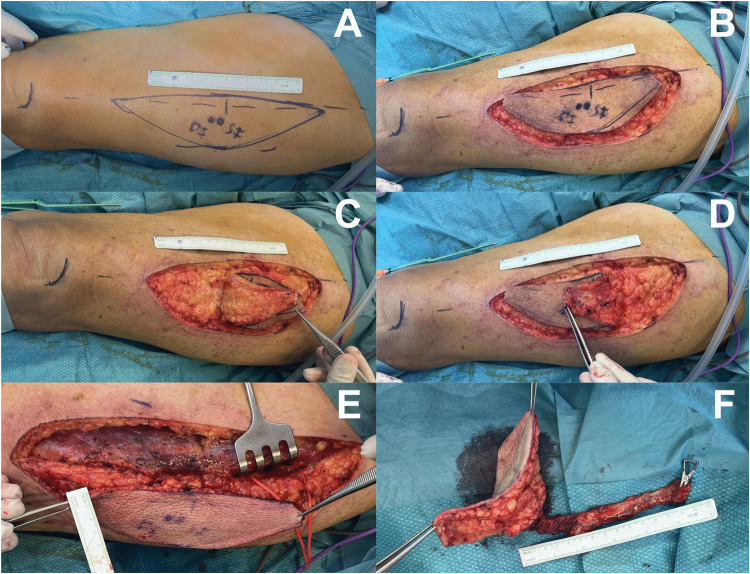
Left super-thin anterolateral thigh flap. On the left side of **A**-**E** figure lateral upper border of the patella is marked. On the right side of the figures is the groin region. **A**: Surgical planning of the flap with marking of the preoperative color Doppler ultrasonographical defined perforator. (SF: the exact spot where the perforator pierces the superficial fascia; DF: the exact spot where the perforator pierces the deep fascia); **B**: Anterior and posterior incision is made till the superficial fascia plane; **C**-**D**: Immediately dissection is made in the superficial fascia plane; **E**: After exploration of the rectus femoris the branch of the lateral circumflex femoral artery is on a vessel loop. The flap thickness is 5–6 mm; **F**: 6 × 11 cm super-thin ALT flap with 15 cm vascular pedicle. Because of the volume of the defect on the floor of the mouth the cuff or tissue left around the perforator above the deep fascia

Using preoperative color Doppler ultrasonography, we define the thickness of the flap as well as the flow rate and course of the perforator (Fig. 1). If the perforator does not run directly to the skin and pierces the deep and superficial fascia at two separate locations, we mark these separately. We plane the flap centered on the perforator. If the perforator is clearly suitable on the preoperative ultrasonography, we immediately dissect the flap in the superficial fascia plane. If not, then using an exploratory incision made on the medial side of the planned flap, we expose the planned perforator subfascial. We analyze it based on its size and pulsation. If there are multiple perforators near each other, we include both in the flap. After preparing the descending branch of the lateral circumflex femoral artery, we dissect the perforator from the septum or muscle. We temporarily close the exploratory incision to create tension for easier dissection in the superficial fascia plane. In accordance with the planned size of the flap, we make an incision on the lateral side of the flap down to the superficial fascia, then we elevate the skin and the superficial fat layer proximally and distally using a Colorado needle monopolar [13, 14]. To ensure safe dissection, leave 2-cm block of tissue from the deep fat layer and the deep fascia around the perforator [10]. If there is a deeper defect in the floor of the mouth, leave a larger margin (Fig. 2). This area fits well onto the floor of the mouth. We trim the flap to the dimensions of the defect and transplant it.

## Results

In the sALT group, oral cavity tumors were included; the clinical T stage of the primary tumor was T1 in one case, T2 in five cases, and T3 in four cases (Table 1). In the RF group, oral cavity and tongue base tumors were included, and the clinical T stage of the primary tumor was T1 in four cases, T2 in 18 cases, T3 in 17 cases, and T4 in three cases.

**Table 1:** Tumor and patient parameter

PN	Tumor localization	Preop th	cT	cN	Sex	Age	Tracheot. ex. POD	NG ex. POD	Inpatient time (day)
1	subl	op, R	2	0	f	79	11	14	17
2	subl	-	2	0	m	51	4	18	28
3	subl	-	3	0	f	60	7	10	14
4	ling	-	3	2b	f	69	4	27	31
5	ling	op	2	2b	m	53	5	14	20
6	subl	-	2	3b	m	70	14	17	22
7	subl	-	1	0	m	66	7	5	46
8	subl	-	3	0	m	71	6	17	25
9	ling	-	2	0	m	26	6	11	15
10	subl	-	3	0	m	58	6	22	24

Abbreviations: *PN* patient number, *subl* sublingual, *ling.* Lingual, *Preop. th* preoperative therapy, *op* surgery, *R* irradiation, *cT* clinical T stadium, *cN* clinical N stadium, *f* female, *m* male, *Tracheot. ex. POD* postoperative day of tracheotomy tube removal, *NG ex. POD* postoperative day of nasogastric tube removal

The tumor localization in the sALT group was presented with seven floor of mouth tumors and three with tongue tumors; in the RFF group were twenty-five floor of mouth tumors and 17 with tongue tumors.

In the sALT group, pathological free margin marginal mandibulectomy was required in 4 cases. Except for four cases, neck dissection was performed in all cases: three patients underwent modified radical or radical neck dissection, while seven patients underwent selective neck dissection. In the RFF group 15 patients underwent marginal or segmental mandibulectomy and in 5 cases neck dissection was not required, in 34 cases selective neck dissection, in 3 cases modified radical neck dissection were performed.

The sALT patients’ body mass index (BMI) ranged from 16 to 31 kg/m^2^, with a mean of 24.8 kg/m^2^, corresponding to a normal weight category. In all cases, color duplex ultrasonography was performed one day preoperatively to locate and mark the perforator vessel, around which the flap was subsequently centered. The BMI of the RFF patients ranged from 18 to 33 kg/m^2^, with a mean of 24.8 kg/m^2^. In all cases, the Allen test was performed one day preoperatively to evaluate the palmar arch.

Tumor resection and flap preparation were carried out in parallel to optimize operation time. Microvascular anastomoses were performed on the neck of all patients. In the sALT group, the superior thyroid artery was used as the recipient artery in 9 cases and the lingual artery in 1 case. For venous outflow, the thyreofacial vein was used in four cases, the external jugular vein in three cases, the internal jugular vein in one case, and the superior thyroid vein in two cases. In the RFF group, the thyroid artery was preferred in 22 cases, lingual artery in 3 cases, and facial artery in 16 cases. For venous outflow, the thyreofacial vein was used in 27 cases, the external jugular vein in 6 cases, the internal jugular vein in 4 cases, and the facial vein in 4 cases. End-to-side anastomosis was performed in the internal jugular vein, whereas end-to-end microsurgical anastomoses with 8–0 or 9–0 sutures were used in all other cases. The sALT perforator flaps were supplied by two perforators in six patients and by a single perforator in four patients (Table 2).

**Table 2:** Patient, flap and surgical parameter

PN	BMI (kg/m^2^)	Flap	Perf. n	Flap thickness (mm)	Pedicle length (cm)	Flap harv. time (min.)	Flap size (cm)	Recip. art	Recip. vein	Surgical time (min.)
1	16.6	ALT	2	5—10	12	124	5.5 × 8	thyrs	thyrs	270
2	23.5	ALT	2	10—15	13	144	6 × 10	ling	thyreof	250
3	25.4	ALT	1	12—18	13	174	6 × 10	thyrs	thyreof	380
4	22.9	ALT	2	8	14	136	6 × 10	thyrs	EJV	306
5	25.7	ALT	1	6	10	137	6 × 10	thyrs	thyreof	345
6	26.7	ALT	2	5—6	15	104	6 × 11	thyrs	EJV	328
7	31.8	ALT	1	10	8	202	5 × 8	thyrs	EJV	340
8	22.3	ALT	2	3—6	10	78	6 × 8	thyrs	thyreof	345
9	21.9	ALT	2	12—15	11	123	6 × 8	thyrs	thyrs	282
10	31.1	ALT	1	10	11	109	7 × 8	thyrs	IJV	285

Abbreviations: *PN* patient number, *BMI* body mass index, *ALT* anterolateral thigh flap, *Perf. n* number of perforators, *flap harv. Time* Flap harvesting time, *Recip. Art.* recipient artery, *Recip. vein* recipient vein, *thyrs* superior thyroid artery, *ling*, lingual artery, *thyreof* thyreofacial vein, *EJV* external jugular vein, *IJV* internal jugular vein, *surgical time* from incision to skin closure

In all sALT cases, circulation of the microsurgical flaps remained stable throughout the follow-up period, and uneventful healing was achieved. As the primary endpoint, (1) sALT is suitable for intraoral reconstruction; (2) we can recruit the sample size recommended by the power analysis.

**Fig. 3:**
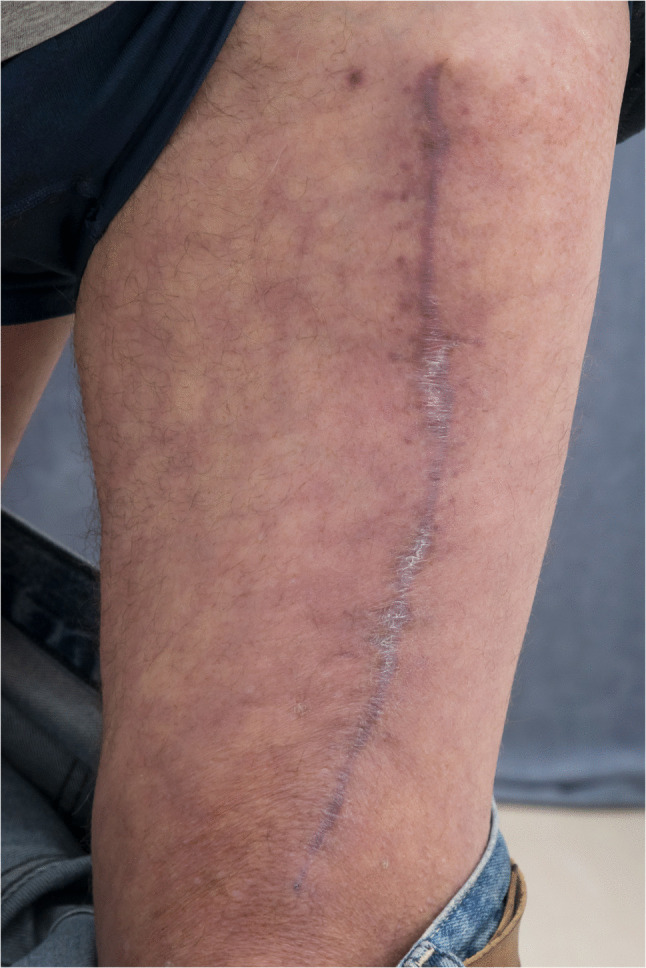
Well healed, primary closure donor side on the left thigh 3 month postoperatively

No donor-site complications such as functional impairment or delayed wound healing were observed (Fig. 3). One patient developed postoperative seroma in the thigh, which resolved after a single puncture. None of the patients reported thigh weakness or walking limitation.

In the RFF group 2 total flap necrosis were observed. The donor-site complication rate for the forearm flap was 14.2% (six cases), most frequently skin necrosis. Half of these cases were treated conservatively, and half required secondary reconstruction with a skin graft. The difference in donor-site complications was not statistically significant (CI: 95%; *p*: 0.81).

Intraoral wound dehiscence (sALT) occurred in three patients, but no postoperative fistula developed. The tracheostomy tube was removed on average on postoperative day 7, and the nasogastric tube was removed on postoperative day 15.5. The mean hospitalization was 24.2 days (Table 1). Intraoral wound dehiscence (RFF) occurred in 3 patients, and a fistula developed postoperatively in 1 patient. The tracheostomy tube was removed on an average on postoperative day 8, and the nasogastric tube on day 12.8. The mean hospitalization was 19.8 days. The difference in tracheostomy (CI: 95%; *p*: 0.589), NG tube (CI: 95%; *p*: 0.337) removal and hospitalization stay (CI: 95%; *p*: 0.182) was not statistically significant (Table 3).

**Table 3:** Statistic of the examined parameters

	95% CI of the Difference		*P* value	Sign
	Lower	Upper		
Tracheostomy removal	−2.69947	4.69947	0.589	NS
NG tube removal	−8.13039	2.83771	0.337	NS
Hospitalization stay	−10.84518	2.11185	0.182	NS
Surfaces	−13.98043	1.96138	0.136	NS
Flap thickness	−3.18111	1.96683	0.610	NS
Pedicle length	-.83587	1.62635	0.522	NS
Donor-site morbidity			0.81	NS

Abbreviations: *NG* nasogastric, *Sign.* Significance, *NS* no significance

**Fig. 4:**
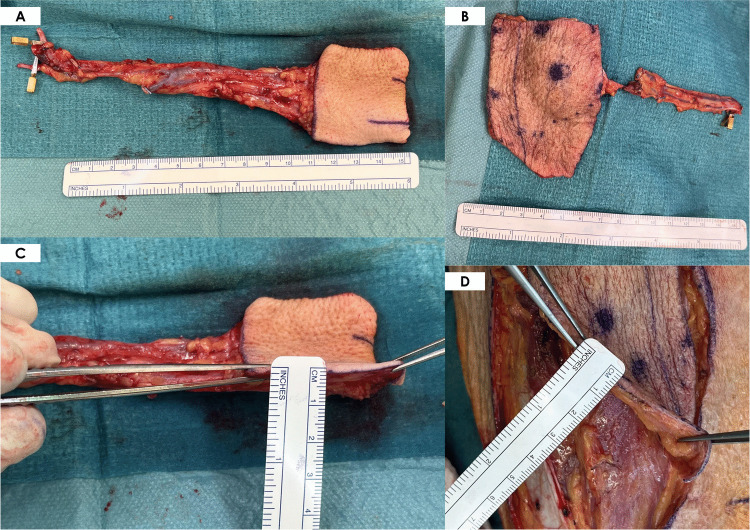
Radial forearm flap and thinned anterolateral thigh flap. **A**: 5 × 5 cm radial forearm flap with 13 cm pedicle length; **B**: 6 × 8 cm anterolateral flap with 11 cm pedicle length, (mind point for perforator); **C**: radial forearm flap with 5 mm flap thickness; **D**: Anterolateral thigh flap with 5 mm flap thickness

The mean operative time for sALT flaps was 313 min (range, 250–380 min) compared with 270 min (range, 160–495 min) for radial forearm flaps. The mean flap surface was 54.2 cm^2^ (range, 40–66 cm^2^; 5.5–7 × 8–10 cm) for sALT and 48.2 cm^2^ (range, 24–72 cm^2^; 3–8 × 6–13 cm) for forearm flaps. The two group of flap surfaces were not significantly different (CI: 95%; *p*: 0.136). The average flap was 9.25 mm (range, 3–18 mm) for sALT and 8,6 mm (range, 5–11 mm) for forearm flaps. The difference in the flap thickness was not statistically significant (CI: 95%; *p*: 0.610). The vascular pedicle length measured 11.7 cm (range, 8–15 cm) for the sALT and 12.1 cm (range, 8–15 cm) for the forearm flap (Fig. 4). The difference in the vascular pedicle was not significant (CI: 95%; *p*: 0.522). Flap harvesting required an average of 133.1 min (range, 78–202 min) for sALT compared with 45.9 min (range, 22–87 min) for the radial forearm flap (Tables 2 and 3).

For the EORTC QLQ-C30/QLQ-H&N35 composite scores, no significant between-group difference was identified preoperatively between the radial forearm free flap (RFFF) and super-thin anterolateral thigh (Super Thin-ALT) flap cohorts (median, 98.0 vs 99.5; Mann–Whitney U = 52.5, *P* = 0.912), and no significant between-group difference was observed postoperatively (median, 112.5 vs 116.0; Mann–Whitney U = 50.0, *P* = 1.000). Within-group paired analysis demonstrated a postoperative increase in composite scores in both reconstructive cohorts, consistent with worse early postoperative status, although these changes did not reach statistical significance with exact Wilcoxon testing (RFFF: median, 98.0 preoperatively vs 112.5 postoperatively; W = 6.0, *P* = 0.055; Super Thin-ALT: median, 99.5 preoperatively vs 116.0 postoperatively; W = 11.0, *P* = 0.105).

For the FACT-H&N composite scores, no significant between-group difference was observed preoperatively between the RFFF and Super Thin-ALT cohorts (median, 77.0 vs 72.0; Mann–Whitney U = 54.0, *P* = 0.796), and no significant postoperative between-group difference was identified (median, 64.5 vs 67.0; Mann–Whitney U = 42.0, *P* = 0.579). In the paired within-group analyses, postoperative FACT-H&N scores were lower than preoperative values in both reconstructive cohorts, indicating worse early postoperative function according to the direction of this instrument’s composite score. This decline reached statistical significance in the RFFF cohort (median, 77.0 preoperatively vs 64.5 postoperatively; Wilcoxon W = 5.0, *P* = 0.039) and showed the same directional pattern in the Super Thin-ALT cohort, without reaching the conventional threshold for significance (median, 72.0 preoperatively vs 67.0 postoperatively; W = 6.0, *P* = 0.055) (Table 4).

**Table 4:** Preoperative and early postoperative evaluation of EORTC QLQ-C30, QLQ-H&N35 and FACT-H&N questionnaires with Mann–Whitney U-test

Instrument	Comparison	Median 1	Median 2	95% CI	P value	Sign
EORTC QLQ-C30/QLQ-H&N35	Preop RFFF vs preop Super Thin-ALT	98.0	99.5	−13.0 to 14.0	.912	NS
EORTC QLQ-C30/QLQ-H&N35	Postop RFFF vs postop Super Thin-ALT	112.5	116.0	−19.0 to 24.0	1.000	NS
EORTC QLQ-C30/QLQ-H&N35	RFFF preop vs postop	98.0	112.5	−0.5 to 44.5	.055	NS
EORTC QLQ-C30/QLQ-H&N35	Super Thin-ALT preop vs postop	99.5	116.0	−7.0 to 43.0	.105	NS
FACT-H&N	Preop RFFF vs preop Super Thin-ALT	77.0	72.0	−7.0 to 12.0	.796	NS
FACT-H&N	Postop RFFF vs postop Super Thin-ALT	64.5	67.0	−21.0 to 5.0	.579	NS
FACT-H&N	RFFF preop vs postop	77.0	64.5	−32.5 to −1.5	**.039**	**S**
FACT-H&N	Super Thin-ALT preop vs postop	72.0	67.0	−18.0 to −1.0	.055	NS

Abbreviations: *preop* preoperative, *postop* postoperative, *RFFF* radial forearm free flap, *ALT* anterolateral thigh flap, *NS* no significance, *S* significance

As a secondary endpoint, (1) there is a difference between the two surgical techniques; (2) a comparable flap can be created from the thigh (sALT) as well as from the forearm (RFF); (3) there is difference in the rate of donor site complications and there is no difference in the quality of life.

## Discussion

**Fig. 5:**
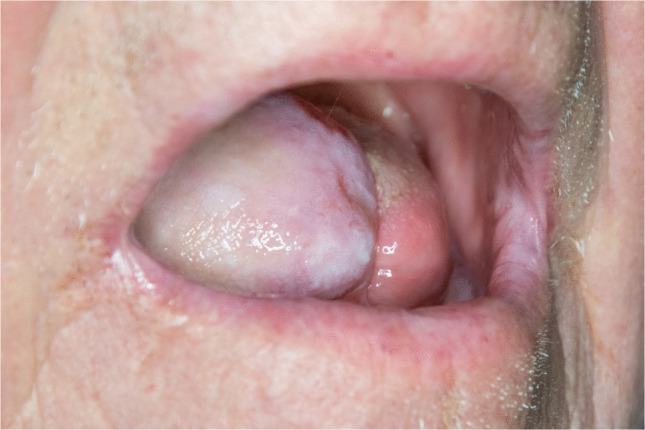
Well healed, intraoral primary closure donor side on the left thigh 3 month postoperatively

Free tissue transfer remains the gold standard for the reconstruction of intraoral defects following resection of locally advanced oral tumors (Fig. 5). The choice of the flap depends primarily on the size and composition of the defect. In cases involving hemiglossectomy and floor of mouth resection, the main objectives are to preserve residual tongue function, achieve reliable closure of the oral cavity to prevent fistula formation, and to facilitate rehabilitation.

The circulation of all sALT flaps was stable. Based on the data obtained, the pilot study was successful with regard to the primary endpoint. Loreti, Hong, Chisolm and Kamizono also concluded that a safe, suitable thinned ALT flap can be harvested from the thigh [13, 15–18]. Many recommendations have been made for preserving and protecting the perforator; the method we have found most effective is to preserve a 2-cm safety zone around the perforator and in this zone the dissection is made with scalpel and scissors [10].

In our national practice, the radial forearm flap is most commonly used because of its thinness, pliability, and reliable blood supply. However, donor-site morbidity associated with this flap is relatively high: (1) the dominant artery of the forearm is sacrificed, (2) grip strength may be reduced, (3) sensory loss can occur in the thumb and index finger, (4) donor sites wider than three centimeters require skin grafting, and (5) grafted skin may result in an esthetically stigmatizing appearance [10]. In contrast, the donor site complication rate of perforator flaps is considerably lower [9, 10, 19].

The anterolateral thigh (ALT) flap, when thinned in the superficial fascia plane (sALT), was originally used mainly for larger soft tissue defects. However, its use has expanded to intraoral reconstruction, offering an alternative to the radial forearm flap, with reduced donor-site morbidity (Table 5). The advantages of sALT include: (1) no sacrifice of the dominant artery, (2) preservation of thigh muscle strength, (3) limited sensory loss in the anterolateral thigh region, (4) primary closure possible for flap widths up to 6–8 cm, and (5) the resulting scar is relatively inconspicuous due to its location and the body contour [20, 21]. Two potential drawbacks of the ALT flap must be emphasized: the variability of its vascular supply and the small caliber of the perforators [22, 23]. Therefore, preoperative color duplex ultrasonography mapping is essential for a reliable flap design. By centering the perforator within the flap, perfusion is optimized, and the risk of vascular kinking or compression at the wound margin is minimized, which is an advantage over the forearm flap in lingual and floor of mouth reconstructions.

**Table 5:** Radial forearm flap and thinned anterolateral thigh flap parameter

	radial forearm flap	super-thin ALT flap
Structure	Skin, subcutis (fascia)	Skin, subcutis
Thickness	5—11 mm	3—18 mm
Pedicle length	8—15 cm	8—15 cm
Vascular diameter	2.0–2.5 mm	2.0 mm
Vascularity	stable	variable
Donor site closure	3 cm < skin graft	6—8 cm > primary closure
Donor site morbidity	Decrease in hand grip strength and numbness of the thumb and index finger	minimal
Ideal size	6 × 8 cm	6 × 12 cm
Surface	24—72 cm^2^	40—66 cm^2^
Average surface	48.2 cm^2^	54.2 cm^2^
Harvesting time	22—87 min	78—202 min

Another distinction lies in the relationship between the flap length and pedicle length. In the radial forearm flap, these parameters were inversely related, with longer flaps associated with shorter pedicles. In contrast, in the sALT flap, the pedicle length was independent of the flap length, as determined by the perforator’s location. Flap harvesting time is influenced by perforator type; intramuscular dissection may prolong the procedure. With increasing surgical experience, the average harvesting time is expected to decrease to 60–90 min. To date, this has not impacted the overall operation time, as flap harvesting is performed in parallel with tumor resection. Similarly, the slightly longer hospital stay observed in our cohort may diminish as more cases accumulate.

As a secondary endpoint, (1) based on the criteria listed above, there is a huge difference between the two surgical techniques; (2) based on mean surface, thickness and pedicle length of the flap, a comparable flap can be created from the thigh (sALT) as well as from the forearm (RFF); (3) there is difference in the rate of donor site complications (sALT 0%, RFF 7.1%) and there is no significant difference in the early quality of life.

Overall, the data indicate that preoperative function was comparable between reconstructive groups and postoperative function was likewise comparable between groups, whereas early postoperative deterioration within groups was evident on both instruments, with the clearest statistical signal seen in the FACT-H&N analysis for the RFFF cohort. This pattern is clinically plausible in the immediate postoperative setting, where speech, swallowing, eating, pain, edema, airway and wound-related issues, and donor-site morbidity commonly worsen short-term patient-reported function and quality of life after oral cancer resection and free-flap reconstruction. Prior literature similarly describes an early postoperative decline in function and well-being, particularly in eating, swallowing, and speech domains, before later recovery in many patients.

Taken together, these findings suggest that flap type was not associated with a measurable difference in early patient-reported functional outcome, whereas the immediate postoperative period was characterized by a decline in function within both reconstructive groups, most evident in the FACT-H&N analysis.

In line with previous articles, we also believe that sALT is associated with a lower rate of donor-site morbidity than RFF [10, 16–18, 24].

The results of this pilot study are subject to several limitations. First the small sample size and the unbalanced groups limits the statistical power to detect significant differences. Second, due to the retrospective nature of the control group design, there is a risk of recall bias. Third, due to the small sample size and retrospective nature of this pilot study (10 vs. 42 patients), statistical adjustments for confounding factors were not feasible. Consequently, these findings should be interpreted as preliminary, and further large-scale prospective studies are required to confirm our results.

Previous articles have discussed recommendations regarding BMI. Kamizono describes the possibility of preparing a similar, thinned ALT for patients with a higher BMI, just as a standard ALT is used for those with a lower BMI [18]. Benanti recommended ALT flap for medium “border” size tumor (50–70 cc) patient under 25 BMI, over 25 the defects is reconstructed with radial forearm flap [25]. Marra recommend standard ALT above 70 cc tumor size [26]. Based on our experience, body type is a much more important factor, than BMI. In our practice, the indication is not determined based on BMI, but rather on a pinch test and color Duplex ultrasonography examination of the thigh. As Vincente published there is no differences in mean speech intelligibility, tongue mobility, or deglutition between ALT and RFF, but ALT is associated with a lower donor site morbidity [24].

In summary, after thorough knowledge of the thigh anatomy and its vascular network, the superficial fascia–thinned ALT flap can serve as a reliable alternative in selected patients to the radial forearm flap for intraoral reconstruction. We recommend that patients with normal and slightly elevated BMI, or thin superficial fat layer on the thigh determined by pinch test or using color duplex ultrasonography can provide comparable functional and esthetic outcomes while reducing donor-site morbidity.

## Conclusion

SALT and RFF showed no significant differences in flap thickness, vascular pedicle, surface, donor site complications, and hospitalization in our pilot study. Superficial fascia–thinned anterolateral thigh flap (sALT) is a safe and reliable option for intraoral reconstruction and did not require additional flap refinement surgeries after reconstruction. It provides functional outcomes comparable to those of the radial forearm flap, while minimizing donor-site morbidity, making it a valuable alternative in selected patients.

## Supplementary Information

Below is the link to the electronic supplementary material.

Supplementary file1 (DOC 99 KB)

Supplementary file2 (DOCX 19 KB)

## Data Availability

No datasets were generated or analysed during the current study.

## Abbreviations

RFF: Radial forearm flap
ALT: Anterolateral thigh flap
sALT: Super-thin anterolateral thigh flap
NG tube: Nasogastric tube
BMI: Body mass index
preop: Preoperative
postop: Postoperative
CI: Confidence interval

## Declarations

### Clinical trial number

BMEÜ/1825–1/2022/EKU.
