# Supplementary material for: Our experience with super-thin anterolateral thigh flap for intraoral reconstruction
Source: Oral Maxillofac Surg. 2026 Aug 12;30(1):130. doi: 10.1007/s10006-026-01616-7 (PMC13461856; doi:10.1007/s10006-026-01616-7)
Supplement: Supplementary file 2 [file 10006_2026_1616_MOESM2_ESM.docx]

**Original article**

**Manuscript title: Our experience with super-thin anterolateral thigh flap for intraoral reconstruction.**

| **Descriptive Statistics RFF** | | | | | |
| --- | --- | --- | --- | --- | --- |
|  | N | Minimum | Maximum | Mean | Std. Deviation |
| lebenyvastagság | 42 | 5.00 | 11.00 | 8.6429 | 1.46206 |
| felület | 42 | 24.00 | 72.00 | 48.1905 | 11.79135 |
| érnyélhossz | 42 | 8.00 | 15.00 | 12.0952 | 1.64997 |
| lebenyelhalás | 42 | .00 | 1.00 | .0476 | .21554 |
| ngex | 41 | 6.00 | 49.00 | 12.8537 | 8.03605 |
| trachex | 40 | 3.00 | 32.00 | 8.0000 | 5.57007 |
| bentfekvés | 42 | 11.00 | 53.00 | 19.8333 | 9.10887 |
| mutetiido | 42 | 160.00 | 495.00 | 270.6667 | 62.01600 |
| Valid N (listwise) | 39 |  |  |  |  |

| **Descriptive Statistics ALT** | | | | | |
| --- | --- | --- | --- | --- | --- |
|  | N | Minimum | Maximum | Mean | Std. Deviation |
| lebenyvastalt | 10 | 4.50 | 15.00 | 9.2500 | 3.56877 |
| érnyélhosszalt | 10 | 8.00 | 15.00 | 11.7000 | 2.11082 |
| feluletalt | 10 | 40.00 | 66.00 | 54.2000 | 8.56089 |
| lebenyelhalasALT | 10 | .00 | .00 | .0000 | .00000 |
| ngexalt | 10 | 5.00 | 27.00 | 15.5000 | 6.24055 |
| trachexalt | 10 | 4.00 | 14.00 | 7.0000 | 3.16228 |
| bentfekvésalt | 10 | 14.00 | 46.00 | 24.2000 | 9.42573 |
| mutetiidőalt | 10 | 250.00 | 380.00 | 313.1000 | 40.96462 |
| Valid N (listwise) | 10 |  |  |  |  |

| **Descriptive Statistics** | | | | | |
| --- | --- | --- | --- | --- | --- |
|  | N | Minimum | Maximum | Mean | Std. Deviation |
| donorhelynecrosisRFF | 42 | .00 | 1.00 | .0714 | .26066 |
| donorhelynecrosisALT | 10 | .00 | .00 | .0000 | .00000 |
| Valid N (listwise) | 0 |  |  |  |  |

| **Independent Samples Test** | | | | | | | | | | | |
| --- | --- | --- | --- | --- | --- | --- | --- | --- | --- | --- | --- |
|  | | Levene's Test for Equality of Variances | | t-test for Equality of Means | | | | | | | |
|  |  | F | Sig. | t | df | Significance | | Mean Difference | Std. Error Difference | 95% Confidence Interval of the Difference | |
|  |  |  |  |  |  | One-Sided p | Two-Sided p |  |  | Lower | Upper |
| lebenyvastagság | Equal variances assumed | 21.952 | <.001 | -.858 | 50 | .198 | .395 | -.60714 | .70771 | -2.02862 | .81433 |
|  | Equal variances not assumed |  |  | -.528 | 9.730 | .305 | .610 | -.60714 | 1.15087 | -3.18111 | 1.96683 |
| érnyélhossz | Equal variances assumed | 1.407 | .241 | .645 | 50 | .261 | .522 | .39524 | .61293 | -.83587 | 1.62635 |
|  | Equal variances not assumed |  |  | .553 | 11.755 | .295 | .590 | .39524 | .71441 | -1.16493 | 1.95541 |
| felület | Equal variances assumed | .604 | .441 | -1.514 | 50 | .068 | .136 | -6.00952 | 3.96847 | -13.98043 | 1.96138 |
|  | Equal variances not assumed |  |  | -1.842 | 18.154 | .041 | .082 | -6.00952 | 3.26179 | -12.85814 | .83909 |
| ngex | Equal variances assumed | .027 | .869 | -.970 | 49 | .168 | .337 | -2.64634 | 2.72896 | -8.13039 | 2.83771 |
|  | Equal variances not assumed |  |  | -1.132 | 17.122 | .137 | .273 | -2.64634 | 2.33870 | -7.57789 | 2.28521 |
| trachex | Equal variances assumed | 1.459 | .233 | .543 | 48 | .295 | .589 | 1.00000 | 1.83995 | -2.69947 | 4.69947 |
|  | Equal variances not assumed |  |  | .750 | 24.917 | .230 | .460 | 1.00000 | 1.33253 | -1.74487 | 3.74487 |
| bentfekvés | Equal variances assumed | .001 | .981 | -1.354 | 50 | .091 | .182 | -4.36667 | 3.22545 | -10.84518 | 2.11185 |
|  | Equal variances not assumed |  |  | -1.325 | 13.303 | .104 | .207 | -4.36667 | 3.29544 | -11.46959 | 2.73626 |
